# Seroprevalence and factors associated with herpes simplex virus type 2 among HIV-negative high-risk men who have sex with men from Rio de Janeiro, Brazil: a cross-sectional study

**DOI:** 10.1186/1471-2334-9-39

**Published:** 2009-04-01

**Authors:** Junia Rodrigues, Beatriz Grinsztejn, Francisco I Bastos, Luciane Velasque, Paula M Luz, Claudia TV de Souza, Ingebourg Georg, Jose H Pilotto, Valdilea G Veloso

**Affiliations:** 1 Instituto de Pesquisa Clínica Evandro Chagas (IPEC), Fundação Oswaldo Cruz, Rio de Janeiro, Brazil; 2 Instituto de Comunicação e Informação Científica e Tecnológica em Saúde (ICICT), Fundação Oswaldo Cruz, Rio de Janeiro, Brazil

## Abstract

**Background:**

Herpes simplex virus type 2 (HSV-2) is the leading cause of genital ulcer disease in developing countries, including Brazil, and is especially prevalent among men who have sex with men (MSM). HSV-2 infection represents a risk factor for the acquisition and transmission of other sexually transmitted diseases. The goal of the present cross-sectional study was to estimate HSV-2 seroprevalence and to determine the factors associated with HSV-2 seropositivity in HIV-negative high-risk MSM from Rio de Janeiro, Brazil.

**Methods:**

Stored sera were tested to estimate HSV-2 seroprevalence, while socio-demographic and sexual behavior data were used to measure associations between risk factors and HSV-2 seropositivity. Using the Poisson regression model with robust variance, prevalence ratios (PR) were used to estimate de degree of association between risk factors and HSV-2 seropositivity in bivariate and multivariate analyses.

**Results:**

Seroprevalence of HSV-2 was of 45.7% (184 out of 403). Factors independently associated with HSV-2 seroprevalence in the multivariate model were: older age (≥ 26 years, PR: 1.41 95% Confidence Interval: 1.11–1.78), non-white race (PR: 1.32 95%CI: 1.06–1.64), positive serology for syphilis (PR: 1.65 95%CI: 1.33–2.05), positive serology for hepatitis B (PR: 1.25 95%CI: 0.99–1.57), stable male partner in the past 6 months (PR: 1.42 95%CI: 1.12–1.79), and unprotected anal sex with a stable female partner (PR: 1.46 95%CI: 1.05–2.04) in the 6 months preceding the cross-sectional assessment.

**Conclusion:**

The present study made evident a high prevalence of HSV-2 infection in a sample of HIV-negative high-risk MSM from Rio de Janeiro. This finding indicates the need and urgency for implementing integrated programs for the prevention of HSV-2 and other sexually transmitted diseases, and, in particular, programs targeting high-risk MSM.

## Background

Herpes simplex virus type 2 (HSV-2) is the leading cause of genital ulcer disease worldwide, and the main etiological agent of genital herpes [1-3]. Although HSV-2 causes anal-genital vesicles and ulcers that can be severe and recurrent, most infections are asymptomatic with intermittent genital excretion of the virus [4-6]. Transmission of HSV-2 to a sexual partner occurs within the whole clinical spectrum of this infection, i.e., in the presence of clinical manifestations, during subclinical episodes and even during periods of asymptomatic virus excretion [7,8]. HSV-2 is most frequently transmitted through the contact with skin or mucosal lesions, or through genital or oral secretions. Although contact with ulcerated lesions is the most common route of transmission, contact with asymptomatic patients can also lead to virus transmission [6,8,9].

HSV-2 infection represents a risk factor for the acquisition and transmission of other sexually transmitted diseases (STDs) [3,10-12]. Observational epidemiological studies have shown that HSV-2 facilitates the acquisition and transmission of human immunodeficiency virus (HIV), a fact that indicates the relevance of the pathogenesis and epidemiology of HSV-2 [2,5,13-16]. Despite its importance, data on the seroprevalence of HSV-2 in different populations are still limited when compared to other STDs, especially in developing countries. Most published data are derived from female populations or from patients seen at STD clinics which are considered to be at higher risk of infection with HSV-2 than the general population [17]. Among men who have sex with men (MSM), reported seroprevalences of HSV-2 vary as a function of HIV status: high (80%) seroprevalences are observed among those co-infected with HIV [18,19] while lower (20%–50%) seroprevalences are reported for those not infected with HIV [20-23].

Significant progress in the understanding of HSV-2 infection has occurred due to the development of highly sensitive and specific serological tests (for the detection of HSV type-specific antibodies) [4]. These tests permit the identification of antibodies specific to HSV-2 [2]. The goal of the present cross-sectional study was to estimate HSV-2 seroprevalence and to determine the factors associated with HSV-2 seropositivity among HIV-negative high-risk MSM from Rio de Janeiro, Brazil.

## Methods

### Study population and study design

A total of 1165 MSM were interviewed at the Evandro Chagas Clinical Research Institute (IPEC), Oswaldo Cruz Foundation, Rio de Janeiro, between January 1994 and December 1998, to evaluate eligibility to the open cohort of HIV-negative individuals ('Projeto Rio', [24]). Cohort eligibility criteria were men aged from 18 to 50 years who engaged in sexual activity with other men and who were HIV-negative at enrollment [25]. 'Projeto Rio' was conceived to estimate important epidemiological measurements, including HIV prevalence and incidence rates, and to prepare local infrastructure for future HIV/STD interventions [24]. The project was funded by the World Health Organization (WHO), the United Nations Joint Program on AIDS (UNAIDS), and the Brazilian Ministry of Health. Additional information on cohort's methods, procedures and aims are available elsewhere [24]. Several studies have been published on data generated through 'Projeto Rio', including HIV incidence rates [25], risk behavior and perception of HIV vulnerability [26], illicit drug use [27], and willingness to participate in HIV vaccine trials [28].

A total of 647 MSM met the eligibility criteria of 'Projeto Rio' thus effectively enrolling in the cohort. At study entry, participants had a blood sample drawn (for HIV, hepatitis B and syphilis testing) and responded to a social and behavioral questionnaire which was administered by a trained psychologist [24]. The present study evaluated HSV-2 seroprevalence for the 403 MSM who first enrolled in 'Projeto Rio'.

### Laboratory procedures

The 403 serum samples obtained at enrollment and stored frozen at the Laboratory of Immunology of IPEC (the original serum bank of 'Projeto Rio') were analyzed by enzyme-linked immunosorbent assay (ELISA) for the detection of IgG class antibodies to HSV-2 glycoprotein G2 (HerpeSelect, Focus Technologies, Cypress, CA). HSV-2 seropositivity was defined by a reactive ELISA with an index ratio ≥ 3.5 in order to increase the specificity of the assay [29-31].

Syphilis testing was performed using two serological assays: the Venereal Disease Research Laboratory (VDRL) test and the *Treponema pallidum *hemagglutination assay (TPHA). Using both assays, two variables were defined: (1) active syphilis when antibody titer with the VDRL test was ≥ 1/8 and the TPHA was positive, and (2) positive serology for syphilis when the VDRL test was not reactive or < 1/8 and TPHA was positive. Positive serology for hepatitis B was assessed by the detection of antibodies to the hepatitis B core antigen (anti-HBC) indicating previous infection.

### Statistical analyses

Factors potentially associated with HSV-2 seropositivity were explored including covariates and confounders previously identified as being related to HSV-2 seropositivity among MSM. Due to the high prevalence of HSV-2 among participants prevalence ratios (PR) were used to estimate the degree of association between HSV-2 infection and potential factors [32]. Using a Poisson regression model with robust variance [32], associations were first evaluated in bivariate analysis. Covariates found to be associated with HSV-2 assuming a threshold level of significance of 0.20 were included in the initial multivariate Poisson regression model. The final multivariate Poisson regression model was reached by stepwise removal of covariates not significantly associated with the outcome, starting with the variable with the highest p-value. Covariates with borderline association for which clinical and/or biological relevance was assumed were kept in the final model. Interactions identified during the modeling process were evaluated in subsequent models; no interaction remained in the final model. Stata Corporation for Windows, version 7.0, was used for the statistical analyses.

### Ethical approval

Written approval was obtained from the Ethics Committee of IPEC, Oswaldo Cruz Foundation, Rio de Janeiro, in order to conduct the HSV-2 serological test on the stored sera. This procedure complies with the National Health Council regulation on research conducted on stored samples.

## Results

The mean and median age of the 403 men was 27.5 (standard error: 7.3 years) and 26 years (interquartile range: 18–49 years), respectively. One hundred and eighty eight (47.2%) men reported 8 years of formal education or less, 251 (66.6%) received 3 minimal-wages or less, and 266 (67.3%) were working (Table 1). Only 38 (9.4%) men reported living with a partner and 339 (84.3%) reported being single.

**Table 1:** Socio-demographic characteristics of the HIV-negative high-risk MSM that participated in the cross-sectional study.

Characteristic	Number* (%)
Age	
< 21	79 (19.6)
21–30	195 (48.4)
> 30	129 (32.0)

Race	
White	205 (52.4)
Non-white	186 (47.6)

Educational level	
≤ 8 years	188 (47.2)
> 8 years	210 (52.8)

Monthly income	
None	50 (13.3)
1–3 minimal wages	201 (53.3)
> 3 minimal wages	126 (33.4)

Working	
No	129 (32.7)
Yes	266 (67.3)

Living with	
Parents or relatives	215 (53.3)
Alone	82 (20.3)
Partner	38 (9.4)
Friend	33 (8.2)
Wife and children	19 (4.7)
Others	16 (4.0)

Marital status	
Single	339 (84.3)
Married	27 (6.7)
Divorced	14 (3.5)
Other	22 (5.5)

*Totals by characteristic vary because of missing values.

One hundred and eighty one men (45.5%) self identified themselves as MSM, while 217 (54.5%) self identified themselves as bisexuals (Table 2). The reported age at first sexual intercourse was less than 16 years for 272 (68.7%) of the men (interquartile range: 12–16 years), the median age at first sexual intercourse did not differ by the gender of the partner. The first sexual intercourse occurred with a woman for 307 (76.2%) of the men. Thirty eight percent of the men reporting sex with women at least once in their lives had their first sex act with another man. More than half of the participants reported having a stable male partner in the past six months (228, 56.6%), and, during this period, unprotected anal sex with a stable male partner was reported by 136 (33.7%) men. Also for the previous six months, 267 (66.3%) men reported having an occasional male partner, and, during this period, unprotected anal sex with an occasional partner was reported by 222 (55.1%) men. Sex with a woman in the past six months was reported by 101 (25.1%) men. Unprotected vaginal sex with a stable and an occasional female partner was reported by 71 (18.3%) and 51 (13.1%) men, respectively. During sexual encounters happening in the past six months, 202 (52.9%) men reported alcohol consumption, while 41 (11.3%) reported cocaine use.

**Table 2:** Behavioral characteristics and prevalence of sexually transmitted diseases among the HIV-negative high-risk MSM that participated in the cross-sectional study.

Characteristic	Number* (%)
Sexual orientation	
Homosexual	181 (45.5)
Bisexual	217 (54.5)

Age at first sexual intercourse	
< 12	82 (20.7)
12–15	190 (48.0)
16–18	90 (22.7)
> 18	34 (8.6)

Age at first sexual intercourse with man	
< 12	33 (13.3)
12–15	76 (30.5)
16–18	83 (33.3)
> 18	57 (22.9)

Age at first sexual intercourse with woman	
< 12	16 (6.3)
12–15	120 (47.2)
16–18	87 (34.3)
> 18	31 (12.2)

First sexual intercourse with	
Man	96 (23.8)
Woman	307 (76.2)

Exchange of sex for money	
Never	228 (56.6)
Sometimes	175 (43.4)

Sex with a woman	
Never	140 (34.7)
Sometimes	263 (65.3)

Sex with a woman in the past 6 months	
Yes	101/403 (25.1)

Occasional male partner in the past 6 months	
Yes	267/403 (66.3)

Stable male partner in the past 6 months	
Yes	228/403 (56.6)

Unprotected anal sex with stable male partner in the past 6 months	
Yes	136/403 (33.7)

Unprotected anal sex with occasional male partner in the past 6 months	
Yes	222/403 (55.1)

Unprotected vaginal sex with stable female partner in the past 6 months	
Yes	71/388 (18.3)

Unprotected anal sex with stable female partner in the past 6 months	
Yes	34/390 (8.7)

Unprotected vaginal sex with occasional female partner in the past 6 months	
Yes	51/390 (13.1)

Unprotected anal sex with occasional female partner in the past 6 months	
Yes	36/389 (9.3)

Alcohol use during sexual encounter in the past 6 months	
Yes	202/382 (52.9)

Cocaine use during sexual encounter in the past 6 months	
Yes	41/362 (11.3)

Herpes simplex virus type 2	
Yes	184/403 (45.7)

Active syphilis**	
Yes	31/403 (7.7)

Positive serology for syphilis**	
Yes	140/403 (34.7)

Positive serology for Hepatitis B***	
Yes	134/403 (33.3)

* Totals by characteristic vary because of missing values. Totals are given next to the number for "No/Yes" dichotomous variables.

** Active syphilis implies that the antibody titer with the VDRL test was ≥ 1/8 and the TPHA was positive, while positive serology for syphilis implies that the VDRL test was not reactive or < 1/8 and TPHA was positive.

*** Positive serology for hepatitis B was assessed by the detection of antibodies to the hepatitis B core antigen (anti-HBC) indicating previous infection.

The point prevalence of HSV-2 was 45.7% (184 out of 403 men, 95% confidence interval: 39.3–53.7%, Table 2). Active syphilis was detected in 31 (7.7%) men and positive serology for syphilis was detected in 140 (34.7%) men. Positive serology for hepatitis B was detected in 134 (33.3%) men.

In the bivariate analysis (Table 3), demographic factors most strongly associated with HSV-2 seropositivity were: older age (≥ 26 years, PR: 1.54 95% Confidence Interval: 1.24–1.92), non-white race (PR: 1.36 95%CI: 1.09–1.70), and living with a partner (PR: 1.41 95%CI: 1.14–1.37). Among behavioral risk factors, younger age at first sexual intercourse with a man (= 15 years PR: 1.28 95%CI: 1.00–1.63), and unprotected anal sex with stable partner in the past 6 months, irrespective of the gender of the partner (PR: 1.20 95%CI: 0.97–1.49 for male partner and PR: 1.46 95%CI: 1.11–1.93 for female partner), were significantly associated with HSV-2 seroprevalence. Positive serology for syphilis (PR: 1.75 95%CI: 1.43–2.15) and hepatitis B (PR: 1.65 95%CI: 1.33–2.05) were also significantly associated with HSV-2 seropositivity.

**Table 3:** Factors associated with herpes simplex virus type-2 seropositivity in the bivariate analysis among the HIV-negative high-risk MSM that participated in the cross-sectional study.

Characteristic	PR	95%CI	p-value
Age			
≤ 26	1.00		
> 26	1.54	1.24–1.92	< 0.001

Race			
White	1.00		
Non-white	1.36	1.09–1.70	0.005

Educational level			
≤ 8 years	1.16	0.93–1.45	0.16
> 8 years	1.00		

Working			
No	1.00		
Yes	1.07	0.84–1.35	0.553

Living with a partner			
Yes	1.41	1.14–1.37	0.001
No	1.00		

Marital status			
Not married	1.00		
Married	1.05	0.96–1.15	0.264

Sexual orientation			
Homosexual	1.00		
Bisexual	1.04	0.83–1.29	0.708

Age at first sexual intercourse			
≤ 15 years	1.21	0.94–1.56	0.122
> 15 years	1.00		

Age at first sexual intercourse with man			
≤ 15 years	1.28	1.00–1.63	0.045
> 15 years	1.00		

Age at first sexual intercourse with woman			
≤ 15 years	1.05	0.82–1.33	0.683
> 15 years	1.00		

First sexual intercourse with			
Man	1.05	0.86–1.39	0.448
Woman	1.00		

Exchange of sex for money			
Never	1.00		
Sometimes	1.09	0.88–1.35	0.407

Sex with a woman			
Never	1.00		
Sometimes	1.08	0.85–1.37	0.499

Sex with a woman in the past 6 months			
No	1.00		
Yes	1.02	0.82–1.28	0.82

Occasional male partner in the past 6 months			
No	1.00		
Yes	1.1	0.87–1.39	0.394

Stable male partner in the past 6 months			
No	1.00		
Yes	1.27	1.02–1.60	0.032

Unprotected anal sex with stable male partner in the past 6 months			
No	1.00		
Yes	1.2	0.97–1.49	0.088

Unprotected anal sex with occasional male partner in the past 6 months			
No	1.00		
Yes	0.86	0.67–1.10	0.236

Unprotected vaginal sex with stable female partner in the past 6 months			
No	1.00		
Yes	1.17	0.90–1.51	0.223

Unprotected anal sex with stable female partner in the past 6 months			
No	1.00		
Yes	1.46	1.11–1.93	0.006

Unprotected vaginal sex with occasional female partner in the past 6 months			
No	1.00		
Yes	0.83	0.57–1.19	0.313

Unprotected anal sex with occasional female partner in the past 6 months			
No	1.00		
Yes	0.7	0.43–1.12	0.142

Alcohol use during sexual encounter in the past 6 months			
No	1.00		
Yes	1.21	0.97–1.52	0.085

Active syphilis*			
No	1.00		
Yes	1.3	0.94–1.79	0.107

Positive serology for syphilis*			
No	1.00		
Yes	1.75	1.43–2.15	< 0.001

Hepatitis B**			
No	1.00		
Yes	1.65	1.33–2.05	< 0.001

* Active syphilis implies that the antibody titer with the VDRL test was ≥ 1/8 and the TPHA was positive, while positive serology for syphilis implies that the VDRL test was not reactive or < 1/8 and TPHA was positive.

** Positive serology for hepatitis B was assessed by the detection of antibodies to the hepatitis B core antigen (anti-HBC) indicating previous infection.

In the multivariate model (Table 4), factors independently associated with HSV-2 seroprevalence were: older age (= 26 years, PR: 1.41 95%CI: 1.11–1.78), non-white race (PR: 1.32 95%CI: 1.06–1.64), positive serology for syphilis (PR: 1.65 95%CI: 1.33–2.05), and hepatitis B (PR: 1.25 95%CI: 0.99–1.57). From the behavioral factors evaluated, those that remained independently associated with HSV-2 seropositivity were stable male partner in the past 6 months (PR: 1.42 95%CI: 1.12–1.79), unprotected anal sex with a stable female partner in the 6 months preceding the baseline interview (PR: 1.46 95%CI: 1.05–2.04).

**Table 4:** Factors associated with herpes simplex virus type-2 seropositivity in the final multivariate model among the HIV-negative high-risk MSM that participated in the cross-sectional study.

Characteristic	PR	95%CI	p-value
Age			
≤ 26	1.00		
> 26	1.41	1.11–1.78	0.004

Race			
White	1.00		
Non-white	1.32	1.06–1.64	0.011

Stable male partner in the past 6 months			
No	1.00		
Yes	1.42	1.12–1.79	0.003

Unprotected anal sex with stable female partner in the past 6 months			
No	1.00		
Yes	1.46	1.05–2.04	0.023

Positive serology for syphilis*			
No	1.00		
Yes	1.65	1.33–2.05	< 0.001

Hepatitis B**			
No	1.00		
Yes	1.25	0.99–1.57	0.053

* Active syphilis implies that the antibody titer with the VDRL test was ≤ 1/8 and the TPHA was positive, while positive serology for syphilis implies that the VDRL test was not reactive or < 1/8 and TPHA was positive.

** Positive serology for hepatitis B was assessed by the detection of antibodies to the hepatitis B core antigen (anti-HBC) indicating previous infection.

## Discussion

The present study made evident a high prevalence of HSV-2 infection (45.7%) in this sample of HIV-negative high-risk MSM from Rio de Janeiro. This result is consistent with other studies conducted among MSM from other countries and in Brazil as well [18,23,33-35]. This finding indicates the need and urgency for implementing integrated programs for the prevention of HSV-2 and other STDs, and, in particular, programs targeting high-risk MSM. HSV-2 is the STD most frequently associated with the genesis of genital ulcer disease in developing countries, including Brazil [3,36,37], and is especially prevalent among MSM [21].

The median age of the studied population was 26 years and chronological age was found to be independently associated with HSV-2 infection. This finding is consistent with published results of other investigators made within the national and international context [4,21,34,38]. The risk of contracting HSV-2 infection shows a practically linear increase with age [39-44], probably reflecting a higher chance of acquiring HSV-2 given a longer exposure time (cumulative exposure) and not an increased susceptibility to infection as a function of age or alterations in immunity as a result of aging, such as observed for other infections/diseases [16,45]. In addition, HSV-2 infection is persistent and, therefore, no spontaneous elimination of the virus occurs [6].

In agreement with the findings of other investigators [1,4,20,21,40,43], non-white race was an independent predictor of HSV-2 seropositivity. This result probably relates to current or historical factors, including racial and ethnic differences associated with poverty, access to health services, behaviors and practices related to health in general and to the sex and age composition of different population strata, and patterns of sexual behavior and affective interaction [1,12]. The high seroprevalence among non-whites strongly suggests that the transmission of HSV-2 infection is influenced by factors other than those related to individual sexual behavior, i.e. by contextual and social factors as mentioned above [40].

Among the variables related to sexual behavior, stable male partner and unprotected anal sex with stable female partner were found associated with HSV-2 seropositivity. These findings could be related to characteristics which are central to the transmission of HSV-2, contributing to the cumulative risk of HSV-2 exposure, which are: repeated exposure in stable affective relationships and the lack of condom use in these stable relationships as repeatedly reported in different behavioral studies [46-48]. The systematic use of condoms is not a common practice among stable partners due to their proximity and intimacy, with protective methods often being considered as impairment to emotional involvement and mutual trust [46-48]. Furthermore, the characteristics of HSV-2 infection, i.e., its chronic and recurrent character [6,8], also contribute to the lack of use of condoms (since, if there is no supposed reason for protection, there is no motivation for the adoption of protective measures).

In the present study, positive serology for syphilis and hepatitis B were associated with HSV-2 seropositivity, and remained independently associated with HSV-2 seropositivity in the multivariate model. Several studies have shown that a history of another (hypothetically previous) STD represents an independent predictor of HSV-2 seropositivity [4,23,40,49]. Specifically with respect to syphilis, positive serology has been independently associated with the acquisition of HSV-2 [41]. The association with syphilis may suggest a residual confounding factor in terms of underlying sexual behaviors and/or HSV-2 infection as a cofactor in syphilis transmission (that is, an inverse association). That is, although the association between HSV-2 infection and other STDs probably reflects common behavioral risk factors, the possibility of biological interactions between different infections also exists [3,12]. It is possible that syphilis is a cofactor associated with a higher excretion of HSV-2 or with a higher susceptibility to the latter infection as documented for HIV infection [10]. However, in the present study the simultaneous analysis of possible risk factors and outcomes prevents any inference regarding the direction and possible temporal sequence of the observed associations.

It is well known that the cross-sectional analysis of data obtained from the baseline interview of a cohort does not allow for the establishment of causal relationships since the reverse causality of the observed associations cannot be ruled out (unless, of course, the reverse association is biologically impossible). However, the central questions of this study mainly benefit from the careful analysis of the statistical associations as long as they are interpreted in view of the pertinent literature.

In the present study behavioral information was obtained through self-report. Social and psychological factors may influence answers given to sensitive questions, such as the sexual behavior and condom use. However, evidence suggests that well-designed questionnaires provide acceptable data when administered in an appropriate manner [50]. In this study, questionnaires were applied by a study psychologist [24].

The population of 403 men included in this study corresponds to only 62% of all eligible men and the findings therefore cannot be extrapolated to all high-risk MSM who fulfilled the inclusion criteria of 'Projeto Rio'. Moreover, although our results are consistent with other studies conducted among MSM [33-35], our findings also do not necessarily apply to the whole MSM population of Rio de Janeiro or Brazil. Indeed, our population is composed of high-risk individuals who were sought out to participate in a prospective HIV seroincidence cohort. It is possible that these first 403 men were at higher risk of HSV-2 when compared to those that later entered the study. If this were true, our results would be over-estimating the HSV-2 seroprevalence. In contrast, it is also possible that the first 403 men represented an inverse selection bias, that is, were indeed more healthy than the remaining participants, and thus our results could be under-estimating the HSV-2 seroprevalence. The basis for this reasoning is the recruitment strategy of the study which sought participants outside the health care clinic environment and independent of clinical symptoms. That is, although the individuals were not ill they agreed to participate in the study, possibly indicating that they were concerned with their health.

The present study analyzes secondary data retrospectively, i.e., the original study was designed with other purposes [24]. Accordingly, a more detailed investigation of specific risk factors for HSV-2 seropositivity, such as the presence or absence of signs or symptoms of herpes in the partner of the participant, the number of partners the participant had throughout life and the occurrence and duration of herpes infection in the participant, was not possible.

However, the present study profits from the use of statistical methods tailored at the high prevalence of the event under analysis. Although the logistic regression model and the calculation of the respective odds ratios are the most frequent approaches used in prevalence studies, in the present investigation we used the Poisson regression model since it allows for the direct estimation of the prevalence ratio. This is the proper approach when the basic assumption underlying the use of logistic models, i.e., the rarity of the event analyzed, is violated. In contrast to the logistic regression model, which overestimates the effective prevalence ratio in these cases, the Poisson model yields adequate estimates. Finally, it was necessary to use a method of robust variance estimation in the application of the Poisson model for better fit [32].

This study made evident a high seroprevalence of HSV-2 among high-risk MSM. This high seroprevalence is a matter of concern for healthcare workers and clinicians, and represents a challenge with respect to the establishment of preventive and control measures. The control and prevention of HSV-2 infection is a growing public health concern because of its infectivity, persistence, and role as a cofactor in the transmission and acquisition of other STDs, including HIV [2,3,5,6,8,10,13]. Also, the costs resulting from HSV-2 infection are significant [11]. Acknowledgment of the burden of HSV-2 is needed in order to appropriately address the control and prevention of this disease and its associated co-morbidities.

## Conclusion

Seroprevalence of HSV-2 among HIV-negative high-risk men was of 45.7%. Factors independently associated with HSV-2 seroprevalence were: older age (= 26 years, PR: 1.41 95% CI: 1.11–1.78), non-white race (PR: 1.32 95%CI: 1.06–1.64), positive serology for syphilis (PR: 1.65 95%CI: 1.33–2.05), positive serology for hepatitis B (PR: 1.25 95%CI: 0.99–1.57), stable male partner in the past 6 months (PR: 1.42 95%CI: 1.12–1.79), and unprotected anal sex with a stable female partner (PR: 1.46 95%CI: 1.05–2.04) in the 6 months preceding the cross-sectional assessment.

## Competing interests

The authors declare that they have no competing interests.

## Authors' contributions

BG, JHP and VGV conceived, designed and coordinated the study, guided the discussion of the results, and drafted the manuscript. JR retrieved the data and participated in the statistical analysis and in the discussion of the results. FIB participated in the study design and in the discussion of the results. LV performed the statistical analysis and participated in the discussion of the results. PML participated in the statistical analysis, in the discussion of the results, and in the writing of the manuscript. CTS was responsible for the cross-sectional data used in this study. IG performed the herpes serologic testing and participated in the interpretation of results. All authors read and approved the final version of the manuscript.

## Pre-publication history

The pre-publication history for this paper can be accessed here:

http://www.biomedcentral.com/1471-2334/9/39/prepub
